# Stress, anxiety, and depression as predictors of daytime sleepiness among Brazilian truck drivers

**DOI:** 10.1192/j.eurpsy.2026.12102

**Published:** 2026-07-31

**Authors:** E. B. Camargo Júnior, R. C. D. Silva

**Affiliations:** University of Rio Verde, Rio Verde, Brazil

## Abstract

**Introduction:**

Truck drivers often experience long work hours, sleep deprivation, and occupational stress, which can contribute to emotional distress and impair sleep quality. Excessive daytime sleepiness (EDS) is a common complaint and may be influenced by symptoms of depression, anxiety, and stress.

**Objectives:**

To examine whether symptoms of stress, anxiety, and depression are significant predictors of excessive daytime sleepiness among Brazilian truck drivers.

**Methods:**

A cross-sectional study was conducted with 597 male truck drivers between April and July 2023 in the municipalities of Formosa and Rio Verde, Goiás, Brazil. Participants completed a structured questionnaire on sociodemographic, occupational, behavioral, and psychosocial characteristics. EDS was assessed using the Epworth Sleepiness Scale (cutoff ≥10). Symptoms of depression, anxiety, and stress were measured using the DASS-21 scale, with the following cutoffs for high risk: depression ≥14, anxiety ≥10, and stress ≥19. Logistic regression analyses (bivariate and multivariate) were performed to identify associations.

**Results:**

Excessive daytime sleepiness was reported by 36.2% of participants. The prevalence of high-risk symptoms was 13.6% for depression, 19.8% for anxiety, and 9.0% for stress. In bivariate analyses, EDS was significantly associated with high-risk levels of stress (OR = 4.03; 95% CI: 2.22–7.30), anxiety (OR = 3.32; 95% CI: 2.19–5.04), and depression (OR = 2.52; 95% CI: 1.56–4.05), all p < 0.001. In the multivariate model, stress (OR = 2.63; 95% CI: 1.37–5.02; p = 0.004) and anxiety (OR = 2.59; 95% CI: 1.62–4.15; p < 0.001) remained significant predictors, while depression was not (p = 0.567).

**Conclusions:**

Symptoms of stress and anxiety were independently associated with greater odds of excessive daytime sleepiness among truck drivers, highlighting the role of mental health in sleep regulation. These findings emphasize the importance of integrating psychosocial assessments into occupational health strategies to promote both mental well-being and road safety in this vulnerable workforce.

**Disclosure of Interest:**

None Declared

